# Synergetic Effect of Plasmonic Gold Nanorods and MgO for Perovskite Solar Cells

**DOI:** 10.3390/nano10091830

**Published:** 2020-09-14

**Authors:** Zhetao Xia, Chenxi Zhang, Zhiying Feng, Zhixing Wu, Zengbo Wang, Xiaohong Chen, Sumei Huang

**Affiliations:** 1 Engineering Research Center for Nanophotonics & Advanced Instrument, Ministry of Education, School of Physics and Electronic Science, East China Normal University, North Zhongshan Rd. 3663, Shanghai 200062, China; 51184700067@stu.ecnu.edu.cn (Z.X.); zhangchenxi@tyut.edu.cn (C.Z.); 52194700020@stu.ecnu.edu.cn (Z.F.); 51194700078@stu.ecnu.edu.cn (Z.W.); xhchen@phy.ecnu.edu.cn (X.C.); 2 School of Electronic Engineering, Bangor University, Bangor LL57 1UT, UK; z.wang@bangor.ac.uk

**Keywords:** perovskite solar cells, Au nanorods, longitudinal plasmon resonance, transverse plasmon resonance, MgO passivation layer, plasmon enhancement

## Abstract

We report new structured perovskite solar cells (PSCs) using solution-processed TiO_2_/Au nanorods/MgO composite electron transport layers (ETLs). The proposed method is facile, convenient, and effective. Briefly, Au nanorods (NRs) were prepared and introduced into mesoporous TiO_2_ ETLs. Then, thin MgO overlayers were grown on the Au NRs modified ETLs by wet spinning and pyrolysis of the magnesium salt. By simultaneous use of Au NRs and MgO, the power conversion efficiency of the PSC device increases from 14.7% to 17.4%, displaying over 18.3% enhancement, compared with the reference device without modification. Due to longitudinal plasmon resonances (LPRs) of gold nanorods, the embedded Au NRs exhibit the ability to significantly enhance the near-field and far-field (plasmonic scattering), increase the optical path length of incident photons in the device, and as a consequence, notably improve external quantum efficiency (EQE) at wavelengths above 600 nm and power conversion efficiency (PCE) of PSC solar cells. Meanwhile, the thin MgO overlayer also contributes to enhanced performance by reducing charge recombination in the solar cell. Theoretical calculations were carried out to elucidate the PV performance enhancement mechanisms.

## 1. Introduction

Perovskite solar cells (PSCs) have made great progress during the recent decade, due to their appropriate band structure, uncomplicated production process, low cost, excellent light absorption, great carrier diffusion length, and ambipolar diffusion [1,2,3]. Since organometal halide perovskites were first introduced by Miyasakaet al. in 2009 [4], the power conversion efficiency (PCE) of PSCs has risen rapidly from 3.8% to current certified 25.2% [5]. To date, a comparable PCE to that of traditional single crystalline or polycrystalline silicon photovoltaic (PV) devices has been realized with PSCs, indicating intense prospects of commercialization.

To promote the PCE of PSC solar cells, vigorous investigators have explored and established diversified device engineering approaches, such as interface modification, morphology optimization, defect and contact passivation, device structure design, light management, etc. [6,7,8,9,10,11,12]. It is well known that the organic–inorganic lead halide perovskites efficiently make use of the visible light spectrum of the solar energy from 350 to 750 nm. However, the usage efficiency of photons with energy out of this electromagnetic wavelength region is impoverished, which restricts the PV performance of PSCs. Therefore, various tactics to promote the light absorption of perovskite semiconductor material over all the visible spectral regions are taken by researchers [10,13]. Effective light management can be accomplished by the reduction of reflection losses at the cell surface and trapping of light in the absorbing layer [14,15]. In the past decade, plasmonic nanostructures have been intensively pursued to improve the efficiency of a wide range of solar cells, including polymer solar cells, dye-sensitized solar cells, and heterojunction solar cells [16,17,18,19].

Recently, researchers have reported that the introduction of insulative metal oxide-coated spherical-shaped noble metal particles into PSC solar cells can promote PV efficiency [11,12,20,21,22,23,24]. The PV performance improvements could be associated with a collective effect of localized surface plasmon resonance (LSPR) promoted light absorption, decreased exciton binding energy [20], plasmonic-induced photon recycling [21], and hot-electron injection [22]. By use of the Fermi golden rule to the plasmon coupling with band electrons, microscopic calculations demonstrate that the device efficiency increase can be induced by the light absorption channel and the reduction of exciton binding energy in PSCs metalized with core−shell nanoparticles. Both absorption and electrical channels of the plasmon PV effect work for device efficiency enhancement [23,24]. To date, however, there is no direct experimental testimony that the PV performance improvement is associated with LSPR-enhanced light absorption. In addition, traditional gold and silver nanoparticles are not efficient in boosting the light absorption of widely reported lead-based perovskite semiconductors, owing to their high absorption coefficient in the wavelength region of 500–650 nm [2,25]. By comparison, perovskite semiconductors bear lower light capture in the lower frequency or longer wavelength region. The absorption coefficient considerably declines with the increase in the wavelength from 650 to 800 nm, and the absorption almost vanishes when the wavelength is longer than 800 nm due to the steep absorption edge [26]. In contrast to the usually used spherical-shaped noble metal nanomaterials, the geometrically anisotropic rod-like structures are likely to present anisotropic conductivity to electron migration between the transverse and longitudinal directions [27]. The transverse resonance absorption peak of rod-like structure can promote the direct employment of the visible light. Longitudinally, it can boost the optical scattering, increase the optical path length, and promote the possibility of photon harvesting, especially for the near-infrared light (NIR) [28]. Therefore, it is expected that the LSPR effect of metal nanorods can effectively utilize photon energy chiefly through both far-field scattering and near-field improvement, thus promoting the PV performance of PSC devices [29]. The investigation of novel plasmonic nanostructures with appropriate and predesigned size and shape is demanded to make this field move forward.

In this contribution, for the first time, we utilize a novel nanostructure of an Au nanorod-MgO overlayer to promote the light-harvesting and PV performance of PSCs. Au NRs were synthesized and introduced onto mesoporous TiO_2_ films. Successively, MgO passivation layers were grown on the gold NR modified mesoporous TiO_2_. Differing from conventional metallic nanospheres, these Au NRs are gifted with strong absorption and scattering efficiencies in a wide wavelength region between 600 and 800 nm. In such structure-engineered TiO_2_/Au NRs/MgO electrodes, the nanorod focuses the incident light, causing effective intensification in both near-field and scattering cross-sections, which enhance the photocurrent of PSCs. Moreover, the thin MgO overlayer also contributes to better PV performance by reducing charge recombination in the device. By embedding Au NRs/MgO in the PSC device, we observed prominent EQE enhancements at the NIR frequencies. As a result, the energy conversion efficiency of the perovskite solar cell increased from 14.7% to 17.4%, demonstrating over 18.3% enhancement, compared with the reference device without modification. The PV performance enhancement mechanisms were investigated using numerical simulations. The theoretical calculations correspond well with our experimental results.

## 2. Materials and Methods

### 2.1. Materials

All chemicals were ordered from Sigma-Aldrich (Shanghai, China) or Alfa Aesar (Shanghai, China), unless otherwise stated. They were used as received without further purification.

### 2.2. Synthesis of Gold Nanorods (Au NRs)

Au NRs were synthesized through a seed-mediated growth approach with slight modification, as previously reported [30]. Specifically, a 500 μL portion of hydrogen tetrachloroaurate-(III) (HAuCl_4_·4H_2_O) aqueous solution (0.01 M) was mixed with 0.7289 g cetyltrimethylammonium bromide (CTAB) in deionized (DI) water (20 mL), and then 1.2 mL of fresh ice-cold NaBH_4_ (0.01 M) solution was rapidly added with gentle mixing for 2 min. The synthetic pale yellow-brown colored solution was kept at 25 °C at least 2 h before the next step, and the obtained seed solution can be stable for a few days.

A 2.0 mL volume of HAuCl_4_ (0.01 M), 0.27 mL of AgNO_3_ (0.01 M), and 1.4578 g of CTAB were mixed in 40 mL of DI water by gentle stirring at 25 °C. After the addition of 0.32 mL of freshly prepared L-Ascorbic acid solution (0.1 M), the growth solution changed from dark yellow to colorless. Subsequently, 96 µL of seed solution was added to the growth solution, and the mixed solution was left quietly at least 6 h at 25 °C. The Au NRs were separated and washed for several times by re-centrifugation at 4000 rpm to remove superfluous CTAB and other possible impurities, and then dispersed in ethanol.

### 2.3. Device Fabrication

Fluorine-doped tin oxide (FTO)-coated slides (Pilkington TEC 15, Xiamen, China) were patterned by etching with Zn powders and 2 M HCl. The etched slides were then cleaned with liquid detergent, acetone, ethyl alcohol, and DI water for 15 min, sequentially, to remove the organic or inorganic residues, and were finally dried in a vacuum oven (Yiheng, Shanghai, China). Isopropyl titanate (200 μL) and anhydrous ethanol (5 mL) were mixed to prepare a clear precursor sol. The formed precursor sol was spin-coated onto the primed FTO substrate at 4500 rpm for 30 s, followed by annealing at 500 °C to form a compact TiO_2_ layer (c-TiO_2_). Diluted TiO_2_ pastes were prepared by mixing TiO_2_ paste (Dyesol 18NR-T, Queanbeyan, Australia) and anhydrous ethanol (weight ratio: 1:3.5), and the resulting pastes were stirred overnight. The mesoporous TiO_2_ (p-TiO_2_) layer was formed on the c-TiO_2_ layer by spin-coating diluted TiO_2_ pastes at 2000 rpm for 45 s. The layer was then sintered at 500 °C for 30 min in air. After the samples cooled down to the room temperature, 75 µL of Au NRs solution (0.20 mg mL^−1^) was first spin-coated onto the p-TiO_2_ film’s surface, followed by heating at 100 °C for 30 min on a hotplate in air. The MgO insulative film was formed on the Au NRs coated p-TiO_2_ layer by spin-coating Mg(CH_3_COO)_2_ dissolved in DI water (0.045 M) at 4000 rpm for 30 s, then heated at 400 °C for 1 h. The perovskite MAPbI_3_ (MA = CH_3_NH_3_) layer was grown by a spin-coating process using a *γ*-butyrolactone (GBL)/dimethylsulphoxide (DMSO) solution of CH_3_NH_3_I and PbI_2_ [6,7]. The perovskite precursor solution was prepared by mixing CH_3_NH_3_I (0.1975 g) powders and PbI_2_ (0.5785 g) in GBL (700 μL) and DMSO (300 μL) at room temperature overnight. The formation of the absorber precursor layers involved spin-coating the perovskite precursor solution at 2000 and 4000 rpm for 20 and 50 s, respectively. At the second spin-coating step, anhydrous chlorobenzene (CB) antisolvent was dripped onto the center of the sample. The perovskite precursor coated sample was heated on a hot plate at 100 °C for 30 min. The hole transport layer (HTL) was deposited by spin-coating a spiro-OMeTAD solution with 0.0723 g spiro-MeOTAD, 28.8 μL of 4-tert-butylpyridine, and 17.5 µL of Li TFSI (520 mg Li TFSI in 1 mL of acetonitrile) dissolved in 1 mL of CB at 4000 rpm for 30 s [31]. Finally, a 100 nm thick Ag electrode with an active area of 0.1 cm^2^ was formed on the Spiro-OMeTAD-coated film by thermal evaporation.

### 2.4. Characterization

The surface morphologies of Au NRs and Au NRs modified p-TiO_2_ films were recorded by a high-resolution field emission scanning electron microscope (FESEM, Hitachi S4800, Tokyo, Japan). The absorption spectra of p-TiO_2_-based perovskite absorbers with various modifications were examined and characterized by means of ultraviolet−visible light (UV−vis) spectrometer (Hitachi, U-3010, Tokyo, Japan). Photoluminescence (PL) spectra were measured using a HORIBA JobinYvon fluoromax-4 fluorescence spectrophotometer (Edison, NJ, USA) with an excitation wavelength of 508 nm at room temperature. The external quantum efficiency (EQE) spectra were recorded with the Optical Power Meter (Newport 2936-R, Irvine, CA, USA). The *J–V* characteristics were measured with the source meter controlled by a computer (Keithley model 2440, Solon, OH, USA) under simulated solar illumination (AM1.5G/100 mW/cm^2^, Newport solar simulator, Irvine, CA, USA) in the air. The electrochemical workstation (PG30.FRA2 Autolab, EcoChemie, Utrecht, The Netherlands) was used to measure electrochemical impedance spectroscopy (EIS) curves of the PSCs.

## 3. Results and Discussion

**Figure 1:**
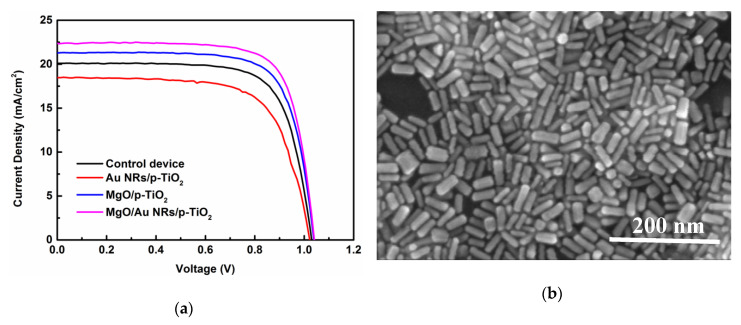

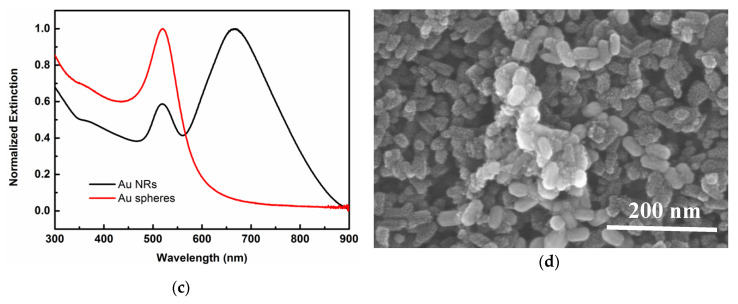
(**a**) *J–V* curves of perovskite solar cell (PSC) devices based on porous TiO_2_ without and with Au NRs and MgO coating. (**b**) FESEM image of as-deposited gold NRs. (**c**) Absorbance spectrum of Au NRs and Au spheres (about 20 nm in diameter) suspensions. (**d**) FESEM image of the top surface morphology of the porous TiO_2_ layer modified with Au NRs.

A series of devices with basic configuration (glass/FTO/c-TiO_2_/p-TiO_2_/CH_3_NH_3_PbI_3_/spiro-OMeTAD/Ag) were fabricated and modified with and without Au NRs and/or MgO. The current density versus voltage (*J–V*) curves from the four types of fabricated PSC devices are shown in Figure 1a. Table 1 lists the summary of PV performance parameters of PSCs, including open-circuit voltage (*V*_OC_), short-circuit current density (*J*_SC_), fill factor (FF), and PCE. Figure 1b,d shows FESEM images of as-deposited gold NRs and the top surface morphology of the porous TiO_2_ layer modified with Au NRs, respectively. From both figures, the average size of Au NRs is about 12 nm in diameter and 40 nm in length, the Au nanorods have an aspect ratio of about 3.3, and Au NRs are distributed on the top surface of the p-TiO_2_ as shown in Figure 1d. The Au nanorods show a transverse SPR band at 519 nm and a longitudinal SPR band at 665 nm, while Au spheres with a diameter of 20 nm display an SPR peak at 520 nm, as shown in Figure 1c.

As can be seen from Table 1, the reference device without Au NRs or MgO coatings shows a PCE value of 14.7% with a *V*_OC_ of 1.02V, *J*_SC_ of 20.10 mA/cm^2^, and FF of 0.72. By incorporating bare Au NRs between the p-TiO_2_ and perovskite layers, the performance of the device degraded notably and displayed a PCE value of 12.7% with a *V*_OC_ of 1.01 V, *J*_SC_ of 18.51 mA/cm^2^, and FF of 0.68. Both short-circuit current density and fill factor values decreased prominently, after the addition of Au NRs alone. Compared to the case of the control device, the device modified by pure MgO exhibited a PCE of 16.4%, the value of *V*_OC_, *J*_SC_, and FF increased a little. The champion cell performance was obtained for the cell modified with both Au NRs and MgO. This cell manifested an open-circuit voltage of 1.04 V, a short-circuit current density of 22.35 mA/cm^2^, and a fill factor of 0.75, resulting in the highest PCE of 17.4%. Compared with the reference device, the PCE enhancement for the device incorporating both Au NRs and MgO mainly comes from the greatly improved short-circuit photocurrent *J*_SC_ (20.10 to 22.35 mA/cm^2^) and the fill factor (0.72 to 0.75), while the open-circuit voltage *V*_OC_ is only slightly changed.

It can be seen that the main performance difference between these four kinds of devices is the short-circuit photocurrent and the fill factor from Table 1. The *J*_SC_ and the fill factor of the PSC device incorporated only Au NPs degraded a lot, which can be associated with two factors. The bared gold NRs can play as charge carrier recombination or trapping sites for photon-induced carriers due to the lower conduction energy level of the Au than that of the TiO_2_ [32,33]. On the other hand, the Au NRs are possibly corroded by the halides during the thermal annealing treatment of the perovskite precursor films, leading to the metal migration or diffusion into the perovskite material, and thus, causing destructive and irreversible alterations to the perovskite active layers and consequently, seriously impacting the solar cell performance [33,34]. From Table 1, the PV performance of the device with pure MgO modification showed enhanced *V*_OC_ values, especially *J*_SC_ and FF values compared with the reference device. The increase in the *V*_oc_ parameter can be chiefly ascribed to the much higher conduction band energy level of MgO than that of TiO_2_. The condition could induce a positive shift of the TiO_2_ conduction band towards that of MgO and achieve a higher quasi-Fermi level under solar light irradiation and a higher open-circuit voltage [35]. Besides, the ultrathin MgO overlayer could play as a tunneling barrier that obstructs the recombination receded from TiO_2_ to the hole transport material in the solar cell, thus, high *J*_sc_ and FF parameters were obtained. Obstruction of charge carrier recombination is also testified by the following electrochemical impedance spectroscopy (EIS) measurement data. As a result, by simultaneously applying Au NRs and MgO coating in the PSC, the solar cell performance was further improved, and the PSC cell using both Au NRs and MgO overlayer is better than the other three kinds of PSC devices. 

As a promising wide bandgap semiconductor and good tunneling and spintronics material, MgO has been widely reported to change the surface state of metal oxide electrodes and reduce interfacial carrier recombination in solar cells [35,36,37]. In our work, in addition to the roles reported in the previous literature, MgO coating also worked as an insulative shell to screen gold nanorods, keeping gold nanorods intact and maintain their structural and thermal stability during the annealing treatment of the perovskite precursor films [33]. The wide bandgap MgO overlayer prevented the gold NRs on the porous TiO_2_ from etching or metal migrating during fabricating perovskite active films. The metal ion diffusion into the absorber layer will have a detrimental effect on device performance and cause leakage current. The MgO coating also blocked the direct contact between gold and the perovskite semiconductor, or the hole conductor material in the PSC, suppressing undesired electron–hole recombination routes within the solar cell. From Table 1, compared with the PSC cell with pure MgO modification, the PSC cell containing both Au NRs and MgO coating displayed analogous *V*_OC_ and FF values, but a remarkably higher short-circuit current. The increase in photocurrent in the latter device compared to the former device suggested that localized surface plasmon resonance (SPR) and electrical effects of Au NRs enhance the PV response of the solar cells [11,38,39,40]. 

**Figure 2:**
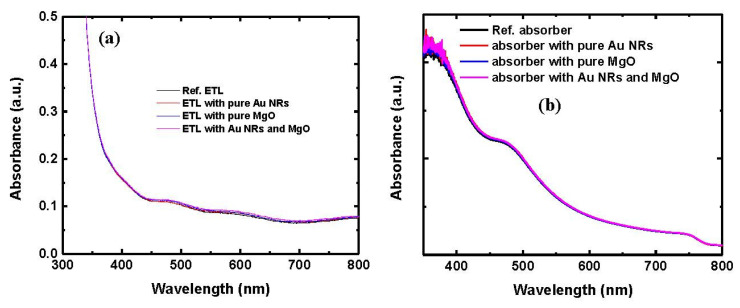

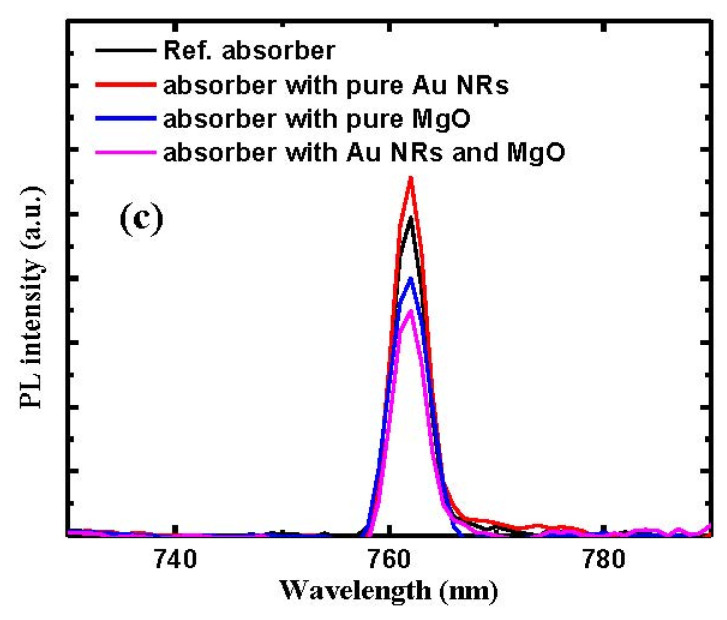
UV–vis absorption of (**a**) p-TiO_2_ electron transport layers (ETLs) and (**b**) perovskite absorbers formed on porous TiO_2_ without and with Au NRs and MgO coating. (**c**) Steady-state photoluminescence (PL) spectra of perovskite absorbers without and with Au NRs and MgO coating.

In order to explore the optical and electrical effects of gold NRs on the PV performance of PSC devices, optical absorption and steady-state PL spectra of the TiO_2_ electron transport layer (ETL) and absorber samples were measured. Figure 2a shows the UV–vis spectra of FTO/c-TiO_2_/p-TiO_2_ samples without or with Au NRs and MgO modification. All the ETL specimens exhibit an analogous light absorption, illustrating that the introduction of Au NRs or MgO into the porous TiO_2_ ETL had no obvious negative effect on the transparency of the TiO_2_ ETL. Figure 2b exhibits the UV–vis spectra of perovskite absorber samples without or with Au NRs and MgO modification. No obvious change was observed in the absorption spectra of the four types of absorber specimen, even in the region of specific absorption wavelengths linked with gold NRs shown in Figure 1c, which is in good agreement with the previously reported results [20,21]. The consequence can be understood by considering the extremely high absorption coefficient of the Pb based perovskite and the small amounts of the added gold NRs or MgO. 

On the other hand, the charge carriers produced in the perovskite absorber of the plasmonic PSC are likely to transport and collect more efficiently. Steady-state PL characterizations are effective in exploring the carrier transporting properties and recombination of light-excited electrons and holes of the solar cells. PL spectra of perovskite absorbers without or with Au NRs and MgO modification are shown in Figure 2c. All absorber samples display an emissive peak at around 761 nm. The PL peak position almost remained constant for the four absorbers, however, their PL intensities and quenching changed a lot. Particularly, the absorber specimen with bare Au NRs displayed the strongest fluorescence signal, and the corresponding perovskite solar cell prospectively showed a higher recombination rate of carriers than the devices based on the other absorber specimens. Nevertheless, compared to the control sample, a significantly enhanced PL quenching was detected for both kinds of absorber samples with MgO overlayer, testifying that electron extraction from perovskite to TiO_2_ ETLs with MgO modification was more efficient than the case to the pristine TiO_2_. Compared with that of the reference absorber without any modification, the fluorescence intensity becomes less and less intense from the absorber with pure MgO to the one containing gold nanorods and MgO. The much lower PL intensity for the latter sample demonstrates that simultaneously incorporating Au NPs and MgO onto porous ETLs plays a dominant role in promoting the charge transfer and blocking the recombination between photogenerated electrons and holes. The perovskite layer grown on the MgO/Au NRs/ETL/FTO substrate displays the most intense PL quenching behavior. The most strongly quenching characteristic suggests the speediest charge transfer, the most efficient electron extraction and the least charge accumulation, and thus, the highest *J*_SC_ value and the best photovoltaic performance [41]. This result is consistent with the PV performance parameter values analyzed in the *J–V* curves of the fabricated PSC devices. 

**Figure 3:**
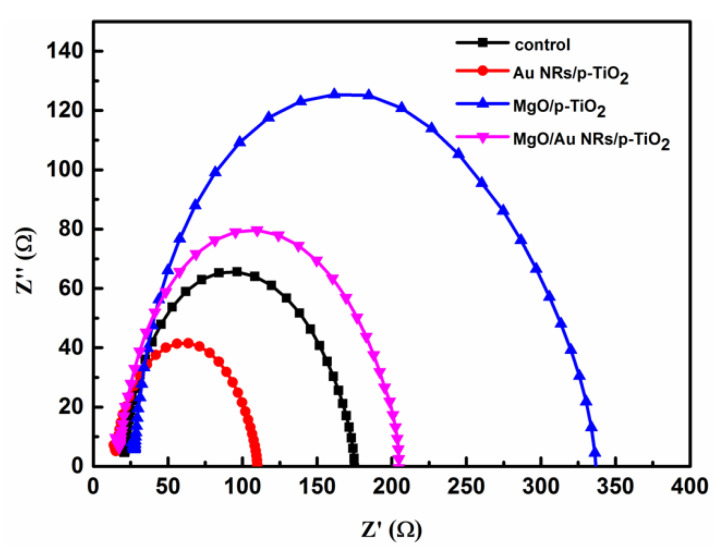
Nyquist plots of PSCs based on p-TiO_2_ without and with Au NRs and MgO overlayer under dark conditions at a 0.9 V applied bias.

EIS characterizations were also performed to study the interface charge transport and recombination in PSC devices. Figure 3 displays the Nyquist plots of the PSCs based on porous TiO_2_ without and with Au NRs or MgO modification. EIS measurements were carried out at a bias voltage of 0.9 V and in the dark. The whole perovskite solar cell can be regarded as a leaking capacitor [42]. The dimension of the semicircle in the Nyquist plot typically symbolizes the magnitude of the recombination resistance at the interface of the ETL/perovskite layer and ETL/hole transport layer. As can be seen from Figure 3, the PSC cell with bare gold nanorods shows the smallest semicircle, indicating the lowest recombination resistance. The control device displays the second-lowest recombination resistance. With the pure MgO modification, the diameter of the semicircle dramatically increases, indicating extremely higher recombination resistance in the device with pure MgO modification than that of the control cell and the PSC with bare Au NRs. The PSC device containing both Au NRs and MgO exhibits the second-largest recombination resistance. Its recombination resistance is a little smaller than that of the PSC with bare MgO. The increase in recombination resistance after using TiO_2_/Au NRs/MgO or TiO_2_/MgO composite ETLs caused a significant decrease in current loss via interfacial carrier recombination, and thus, promoted the *J*_SC_ and the FF parameters for the corresponding PSCs as shown in Table 1.

**Figure 4:**
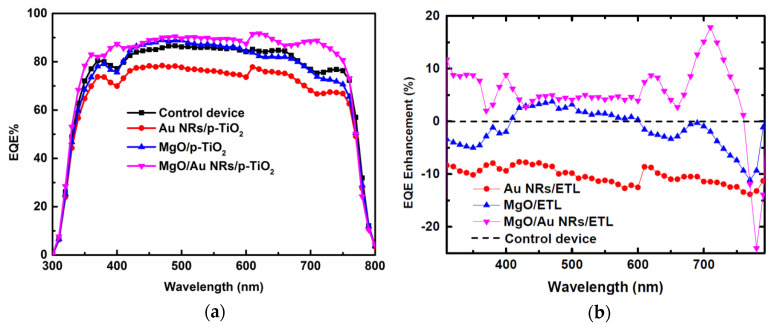
(**a**) External quantum efficiency (EQE) curves of the control (reference) device and the PSCs with Au NRs or MgO overlayer. (**b**) EQE enhancements of PSCs with Au NRs and MgO overlayer: EQE enhancement: ΔEQE/EQE_ref_ (ΔEQE, the difference between EQE values of the modified and the reference devices).

EQE spectra were characterized to further investigate the photoelectric conversion capacity in the modified PSCs. The EQE can be factorized into three components enabling the origin of photocurrent loss to be examined. The element is related to the efficiency of photon harvesting by the perovskite absorber, charge injection from the excited absorber to the TiO_2_ ETL, and charge collection from the TiO_2_ electrode to the external circuit [43]. Thus, it is more appropriate than *J–V* measurements for probing optical and electrical responses of PSCs. The EQE spectra of PSCs modified with and without Au NRs and/or MgO are shown in Figure 4a. From the figure, the generation of photocurrent begins at ∼800 nm, corresponding with the bandgap of perovskite CH_3_NH_3_PbI_3_. In order to examine the positive contribution of gold NRs/or magnesium oxide to the photocurrent, relative EQE variations are exhibited in Figure 4b for different wavelengths. For an incident photon with a wavelength (λ), when the relative EQE enhancement is positive, the presence of the modification improved the efficiency of the device, compared to the unmodified reference device. Negative EQE variation values mean that fewer photons were absorbed or fewer charge carriers were separated and collected by the modified PSC device. It is found that the relative EQE variation for the PSC using bare Au NRs is negative over the wavelength region from 310 to 790 nm. In this case, the presence of bare NRs is actually deleterious to the performance of the PSC device, yielding PV cells with lower power generation than the references in the spectral range. The negative performance observed for bare Au NRs has been attributed to the reasons that the bared gold NRs can work as charge carrier recombination or trapping sites for photon generated carriers and cause destructive alterations to perovskite absorbers. The obtained EQE result for the PSC using bare Au NRs corresponds well with the decrease in the *J*_SC_ data given in Table 1. EQE of the PSC with pure MgO modification was clearly improved in the wavelength region from about 400 to 600 nm when compared with that of the reference device. In contrast, for the PSC cell containing both Au NRs and MgO overlayer, the relative EQE variation curve presents positive values almost in the whole spectral range from 310 to 750 nm. The EQE enhancements are dominant at long wavelengths, significantly displaying two positive maxima at 620 nm and 709 nm, respectively. The characteristic EQE enhancement profile closely matches the Au NR extinction spectrum in Figure 1c. The positions of the maxima in long wavelengths could be associated with the longitudinal SPR bands of the colloid Au NRs immobilized on Au- and MgO-modified PSC devices. The positive EQE enhancement values could be partly attributed to the favorite radiation scattering into the PSC absorber mediated by the local SPR [18]. In the case of simultaneous inclusion of Au NRs and MgO coating, the incident radiation and the scattering light from colloidal Au NRs lead to constructive interference of the light waves combined at the interface of the perovskite absorber and TiO_2_ ETL. Additionally, the EQE enhancement can be also ascribed to the hole blocking effect of MgO, which reduced the charge recombination in the modified device. Although multipolar scattering, such as quadrupolar contributions, can be very efficient [44], their effect at shorter wavelengths is very complicated in consideration of the mechanisms of interband transition. From Figure 4a,b, the trend in EQE enhancement for differently structured PSC devices agrees well with ours with that from *J–V* measurement and analysis shown in Figure 1a and Table 1.

**Figure 5:**
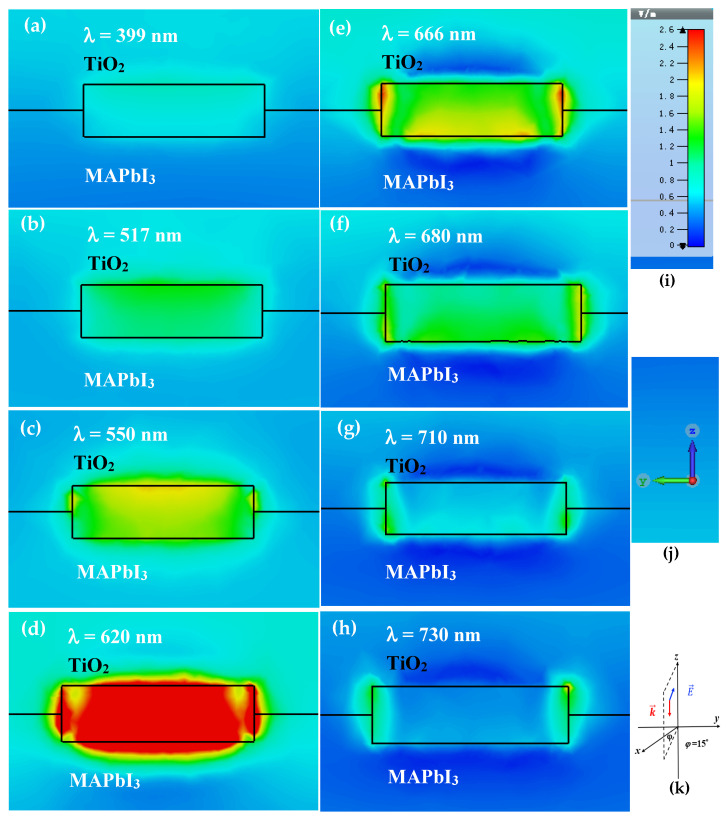
(**a**–**h**) Simulated field |E| enhancement factor distribution around Au NR (40 nm in length and 11.6 nm in diameter) at different wavelengths, (**i**) color scale and (**j**) *yz* plane. The local field-enhancement factor distributions on *yz* planes at *x* = 0 nm. (**k**) Incident light beam propagation is along −*z*, and the light polarized direction.

To better understand the optical effect of Au NRs on the PV performance, Finite-difference time-domain (FDTD) numerical simulations were performed to simulate the electromagnetic field within the perovskite layer containing Au NRs. The finite integral technique (CST Microwave Studio) was applied for the calculations [12,33,45]. The optical properties (refractive index, *n*, and extinction coefficient, *k*) of the related materials are obtained from previous publications [25,46,47]. FDTD numerical simulations were performed for an Au naorod (40 nm in length and 11.6 nm in diameter) embedded between TiO_2_ and perovskite layers. The Au nanorod is along the *y*-direction. The incident light is a plane wave propagating at the normal incidence along the negative *z*-direction, and the polarization direction is shown in the bottom-right corner of Figure 5. The electric-field |E| enhancement factor distributions around the Au NR embedded between TiO_2_ and perovskite layers at different wavelengths are exhibited in the figure. The enhancement factor is determined as the proportion between the electric field within the TiO_2_ or perovskite layers with and without the plasmonic NRs. As can be seen from Figure 5, the near-field enhancement factor around Au NR is noticeable and changes with the wavelength of the incident light. The most prominent augmentation typically occurs at the edge of the Au nanorod. The |E| enhancement factor of the local field is extremely high and the strength dimension is most considerably large at 620 nm. The near-field enhancement can be a result of coupling between individual plasmonic Au NRs and the interaction between Au NR and TiO_2_/CH_3_NH_3_PbI_3_ in close proximity [48]. When metal and semiconductor nanomaterials are very adjacent to each other, higher-order interactions and even energy transfer may occur [49]. 

**Figure 6:**
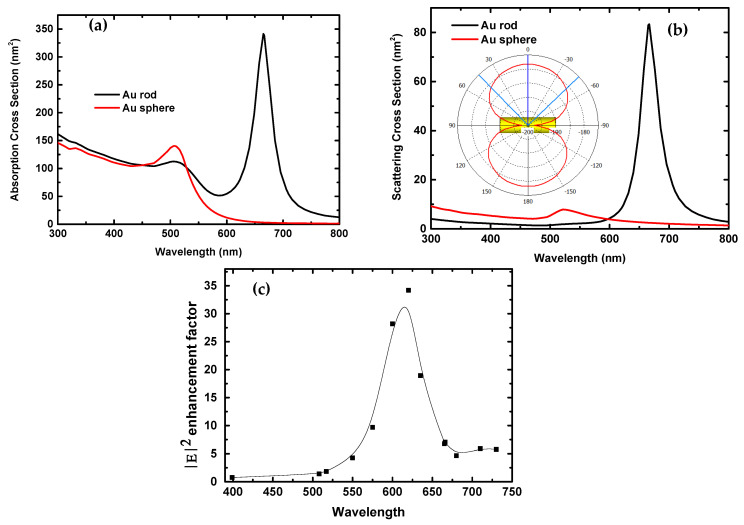
Calculated absorption (**a**) and scattering (**b**) cross-sections of an Au NR (40 nm in length and 11.6 nm in diameter) and sphere (20.06 nm in diameter) by a linear polarized plane wave in free space. The insert is the scattering diagram for the Au NR at a wavelength of 620 nm. (**c**) Maximal field intensity |E|^2^ enhancement factor around the Au NR at different wavelengths.

Precious metal NRs are exceptionally appealing for their high absorption and scattering efficiencies in the NIR frequencies. The corresponding longitudinal plasmon resonances (LPRs) are sensitive to the polarization of the incident excitation. Figure 6a,b shows the calculated absorption and scattering cross-sections of an Au naorod (40 nm in length and 11.6 nm in diameter) and an Au sphere (20.06 nm in diameter) with the same volume as the gold nanorod. The polarization direction of the incident plane wave is the same as shown in Figure 5. The nanoorod is along the y-direction. From the simulation, gold nanorods support tremendously higher absorption and scattering cross-sections than Au spheres with the same volume as the Au NRs at NIR wavelengths (600–800 nm). Additionally, for the Au NR, the absorption cross-section value is higher than the scattering cross-section value at a fixed wavelength. The results are in good agreement with the previous work [50,51]. The insert in Figure 6b shows an angular scattering diagram for the Au nanorod illuminated by a linear polarized plane wave with a wavelength of 620 nm in free space. Furthermore, the simulated transverse and longitudinal plasmon resonances (LPSs) of the Au NRs are 508 nm and 665 nm, respectively, from the absorption cross-section curve shown in Figure 6a. The scattering cross-section curve also displays an LPS at 665 nm, as shown in Figure 6b. The calculated transverse plasmon resonance and LPS results are very consistent with those from the measured absorbance spectra of Au NRs and spheres shown in Figure 1c. 

Figure 6c shows the dependence of the maximal electric-field intensity |E|^2^ enhancement factor around Au NR embedded between TiO_2_ and MAPbI_3_ films on the wavelength of the incident light beam. From the figure, the maximal |E|^2^ enhancement factor increases with the increase in the wavelength from 400, reaches a maximum at 620 nm, and then drops down with the further increase in the wavelength from 620 nm. The electric field’s intensity in the perovskite layer containing Au NRs can be increased by close to 35 times than the incident light field at the wavelength of 620 nm, where both the maximal absolute EQE value and one EQE enhancement peak took place as shown in Figure 4a,b, respectively. This coincidence between the near-field enhancement peak and the position of the maximal absolute EQE or the positive peak in EQE variations undoubtedly support that the enhanced local electric field boosted the generation rate of electron–hole pairs and thereby upgraded the PV performance of PSC solar cells followed by increases in photocurrent and power conversion efficiency [21]. In our study, the nanorods are randomly distributed in the perovskite absorber, which induces complexity in the effecting of LSPRs, but still will evidently enhance the optical response and the EQE values at plasma resonances.

We have mainly discussed the optical effect of Au NRs on the PV performance in the above simulation sections. Actually, plasmons can contribute to modifying not only light absorption but also local electricity inside a PSC cell metalized with core−shell nanoparticle [12,20,21,22,23,24]. Both optical and electrical channels of plasmon mediation impact the device PV performance with the eventual value being the subject of the trade-off between competing factors in complicated dependence of size, shape, component, and material parameters of metal nanostructures and absorber semiconductors. Microscopic calculations demonstrated that nonvertical interband transitions provide an effect unrelated to photoabsorption, nevertheless, which can promote the device efficiency by adjusting its internal electricity [23]. This electrical channel of the plasmon PV effect is ascribed to the decrease in the exciton binding energy, which assists the progress of exciton dissociation at the interface with the TiO_2_ porous electrode of a plasmonic PSC, resulting in the device PV performance enhancement. 

## 4. Conclusions

We have investigated the synergistic effect of Au NRs and MgO on the PV performance of CH_3_NH_3_PbI_3_ based PSCs. By the simultaneous use of Au NRs and MgO, the power conversion efficiency of the PSC device increases from 14.7% to 17.4%, displaying an over 18.3% enhancement, compared with the reference device without modification. It was found that the incorporated Au NRs increased the absorption cross-section and scattering cross-section of the incident light, and prominently promoted the near-field and the scattering efficiency chiefly by longitudinal plasmon resonances of gold NRs. Moreover, the presence of MgO significantly increases the *V*_OC_ of the device by reducing charge recombination. The aforementioned conclusions are revealed by *J–V* curves, PL spectroscopy, EIS spectrum, and EQE analysis. As the advantage of Au NR and MgO can be well-employed in this study, the photovoltaic performance of the PSC cells was significantly enhanced. The study suggests that the simultaneous use of Au NRs and MgO overlayer is an effective route in developing high-performance perovskite-based photoelectric devices.

## Figures and Tables

**Table 1:** Photovoltaic parameters of the PSCs based on m-TiO_2_ with and without Au nanorods (NRs) and/or MgO.

Parameters	*V*_OC_ (V)	*J_SC_* (mA/cm^2^)	FF	PCE (%)
Control	1.02	20.10	0.72	14.7
With pure Au NRs	1.01	18.51	0.68	12.7
With pure MgO	1.04	21.30	0.74	16.4
With Au NRs/MgO	1.04	22.35	0.75	17.4

## References

[1] Huang J., Yuan Y., Shao Y., Yan Y. (2017). Understanding the physical properties of hybrid perovskites for photovoltaic applications. Nat. Rev. Mater..

[2] Green M.A., Ho-Baillie A., Snaith H.J. (2014). The emergence of perovskite solar cells. Nat. Photonics.

[3] Yang L., Wang X., Mai X., Wang T., Wang C., Li X., Murugadoss V., Shao Q., Angaiah S., Guo Z. (2019). Constructing efficient mixed-ion perovskite solar cells based on TiO_2_ nanorod array. J. Colloid Interface Sci..

[4] Kojima A., Teshima K., Shirai Y., Miyasaka T. (2009). Organometal halide perovskites as visible-light sensitizers for photovoltaic cells. J. Am. Chem. Soc..

[5] NREL Best Research-Cell Efficiency Chart. https://www.nrel.gov/pv/cell-efficiency.html.

[6] Jeon N.J., Noh J.H., Kim Y.C., Yang W.S., Ryu S., Seok S.I. (2014). Solvent engineering for high-performance inorganic–organic hybrid perovskite solar cells. Nat. Mater..

[7] Zhang C., Luo Y., Chen X., Chen Y., Sun Z., Huang S. (2016). Effective improvement of the photovoltaic performance of carbon-based perovskite solar cells by additional solvents. Nano-Micro Lett..

[8] Jiang Q., Zhao Y., Zhang X., Yang X., Chen Y., Chu Z., Ye Q., Li X., Yin Z., You J. (2019). Surface passivation of perovskite film for efficient solar cells. Nat. Photonics.

[9] Chi W., Banerjee S.K. (2020). Progress in materials development for the rapid efficiency advancement of perovskite solar cells. Small.

[10] Kakavelakis G., Petridis K., Kymakis E. (2017). Recent advances in plasmonic metal and rare-earth-element upconversion nanoparticle doped perovskite solar cells. J. Mater. Chem. A.

[11] Moakhar R.S., Gholipour S., Masudy-Panah S., Seza A., Mehdikhani A., Riahi-Noori N., Tafazoli S., Timasi N., Lim Y.F., Saliba M. (2020). Recent advances in plasmonic perovskite solar cells. Adv. Sci..

[12] Luo Q., Zhang C., Deng X., Zhu H., Li Z., Wang Z., Chen X., Huang S. (2017). Plasmonic effects of metallic nanoparticles on enhancing performance of perovskite solar cells. ACS Appl. Mater. Interfaces.

[13] Xu Q., Zhao Y., Zhang X. (2020). Light management in monolithic perovskite/silicon tandem solar cells. Sol. RRL.

[14] Sun S., Salim T., Mathews N., Duchamp M., Boothroyd C., Xing G., Sum T.C., Lam Y.M. (2014). The origin of high efficiency in low-temperature solution-processable bilayer organometal halide hybrid solar cells. Energy Environ. Sci..

[15] Wang H.-P., Lien D.-H., Tsai M.-L., Lin C.-A., Chang H.-C., Lai K.-Y., He J.-H. (2014). Photon management in nanostructured solar cells. J. Mater. Chem. C.

[16] Lee J.H., Park J.H., Kim J.S., Lee D.Y., Cho K. (2009). High efficiency polymer solar cells with wet deposited plasmonic gold nanodots. Org. Electron..

[17] Ding B., Lee B.J., Yang M., Jung H.S., Lee J.K. (2011). Surface-plasmon assisted energy conversion in dye-sensitized solar cells. Adv. Energy Mater..

[18] Wang P.H., Millard M., Brolo A.G. (2014). Optimizing plasmonic silicon photovoltaics with Ag and Au nanoparticle mixtures. J. Phys. Chem. C..

[19] Chen Y., Li Z., Chen X., Liu C., Ye X., Wang Z., Sun Z., Huang S. (2012). Improved performance of flexible amorphous silicon solar cells with silver nanowires. J. Appl. Phys..

[20] Zhang W., Saliba M., Stranks S.D., Sun Y., Shi X., Wiesner U., Snaith H.J. (2013). Enhancement of perovskite-based solar cells employing core–shell metal nanoparticles. Nano Lett..

[21] Saliba M., Zhang W., Burlakov V.M., Stranks S.D., Sun Y., Ball J.M., Johnston M.B., Goriely A., Wiesner U., Snaith H.J. (2015). Plasmonic-induced photon recycling in metal halide perovskite solar cells. Adv. Funct. Mater..

[22] Yuan Z., Wu Z., Bai S., Xia Z., Xu W., Song T., Wu H., Xu L., Si J., Jin Y. (2015). Hot-electron injection in a sandwiched TiO_x_–Au–TiO_x_ structure for high-performance planar perovskite solar cells. Adv. Energy Mater..

[23] Jacak W.A., Jacak J.E. (2019). New channel of plasmon photovoltaic effect in metalized perovskite solar cells. J. Phys. Chem. C..

[24] Laska M., Krzemińska Z., Kluczyk-Korch K., Schaadt D., Popko E., Jacak W., Jacak J. (2020). Metallization of solar cells, exciton channel of plasmon photovoltaic effect in perovskite cells. Nano Energy.

[25] Ball J.M., Stranks S.D., Hörantner M.T., Hüttner S., Zhang W., Crossland E.J., Ramirez I., Riede M., Johnston M.B., Friend R.H. (2015). Optical properties and limiting photocurrent of thin-film perovskite solar cells. Energy Environ. Sci..

[26] Tan H., Jain A., Voznyy O., Lan X., De Arquer F.P.G., Fan J.Z., Quintero-Bermudez R., Yuan M., Zhang B., Zhao Y. (2017). Efficient and stable solution-processed planar perovskite solar cells via contact passivation. Science.

[27] Wang Y., Zhou X., Liang C., Li P., Hu X., Cai Q., Zhang Y., Li F., Li M., Song Y. (2017). Enhanced efficiency of perovskite solar cells by using core–ultrathin shell structure Ag@ SiO_2_ nanowires as plasmonic antennas. Adv. Electron. Mater..

[28] Gangadharan D.T., Xu Z., Liu Y., Izquierdo R., Ma D. (2017). Recent advancements in plasmon-enhanced promising third-generation solar cells. Nanophotonics.

[29] Chan K., Wright M., Elumalai N., Uddin A., Pillai S. (2017). Plasmonics in organic and perovskite solar cells: Optical and electrical effects. Adv. Opt. Mater..

[30] Nikoobakht B., El-Sayed M.A. (2003). Preparation and growth mechanism of gold nanorods (NRs) using seed-mediated growth method. Chem. Mater..

[31] Jiang Z., Chen X., Lin X., Jia X., Zhuo S. (2016). Amazing stable open-circuit voltage in perovskite solar cells using AgAl alloy electrode. Sol. Energy Mater. Sol. Cells.

[32] Subramanian V., Wolf E.E., Kamat P.V. (2004). Catalysis with TiO_2_/gold nanocomposites. Effect of metal particle size on the Fermi level equilibration. J. Am. Chem. Soc..

[33] Zhang C., Luo Q., Shi J., Yue L., Wang Z., Chen X., Huang S. (2017). Efficient perovskite solar cells by combination use of Au nanoparticles and insulating metal oxide. Nanoscale.

[34] Domanski K., Correa-Baena J.P., Mine N., Nazeeruddin M.K., Abate A., Saliba M., Tress W., Hagfeldt A., Grätzel M. (2016). Not all that glitters is gold: Metal-migration-induced degradation in perovskite solar cells. Acs Nano.

[35] Wang J., Qin M., Tao H., Ke W., Chen Z., Wan J., Qin P., Xiong L., Lei H., Yu H. (2015). Performance enhancement of perovskite solar cells with Mg-doped TiO_2_ compact film as the hole-blocking layer. Appl. Phys. Lett..

[36] Guo X., Dong H., Li W., Li N., Wang L. (2015). Multifunctional MgO layer in perovskite solar cells. ChemPhysChem.

[37] Dagar J., Castro-Hermosa S., Lucarelli G., Cacialli F., Brown T.M. (2018). Highly efficient perovskite solar cells for light harvesting under indoor illumination via solution-processed SnO_2_/MgO composite electron transport layers. Nano Energy..

[38] Stratakis E., Kymakis E. (2013). Nanoparticle-based plasmonic organic photovoltaic devices. Mater. Today.

[39] Wang D.H., Kim D.Y., Choi K.W., Seo J.H., Im S.H., Park J.H., Park O.O., Heeger A.J. (2011). Rücktitelbild: Enhancement of donor–acceptor polymer bulk heterojunction solar cell power conversion efficiencies by addition of Au nanoparticles. Angew. Chem. Int. Ed..

[40] Jang Y.H., Jang Y.J., Kim S., Quan L.N., Chung K., Kim D.H. (2016). Plasmonic solar cells: From rational design to mechanism overview. Chem. Rev..

[41] Zhu G., Lin T., Lü X., Zhao W., Yang C., Wang Z., Yin H., Liu Z., Huang F., Lin J. (2013). Black brookite titania with high solar absorption and excellent photocatalytic performance. J. Mater. Chem. A.

[42] Boix P.P., Larramona G., Jacob A., Delatouche B., Mora-Seró I., Bisquert J. (2012). Hole transport and recombination in all-solid Sb_2_S_3_-sensitized TiO_2_ solar cells using CuSCN as hole transporter. J. Phys. Chem. C.

[43] Barnes P.R., Anderson A.Y., Koops S.E., Durrant J.R., O’Regan B.C. (2009). Electron injection efficiency and diffusion length in dye-sensitized solar cells derived from incident photon conversion efficiency measurements. J. Phys. Chem. C.

[44] Morawiec S., Mendes M.J., Mirabella S., Simone F., Priolo F., Crupi I. (2013). Self-assembled silver nanoparticles for plasmon-enhanced solar cell back reflectors: Correlation between structural and optical properties. Nanotechnology.

[45] Fan W., Yan B., Wang Z., Wu L. (2016). Three-dimensional all-dielectric metamaterial solid immersion lens for subwavelength imaging at visible frequencies. Sci. Adv..

[46] Liu L., Zhong H., Bai Z., Zhang T., Fu W., Shi L., Xie H., Deng L., Zou B. (2013). Controllable transformation from rhombohedral Cu_1.8_S nanocrystals to hexagonal CuS clusters: Phase-and composition-dependent plasmonic properties. Chem. Mater..

[47] Johnson P.B., Christy R.-W. (1972). Optical constants of the noble metals. Phys. Rev. B.

[48] Wintzheimer S., Granath T., Oppmann M., Kister T., Thai T., Kraus T., Vogel N., Mandel K. (2018). Supraparticles: Functionality from uniform structural motifs. ACS Nano.

[49] Li J., Cushing S.K., Meng F., Senty T.R., Bristow A.D., Wu N. (2015). Plasmon-induced resonance energy transfer for solar energy conversion. Nat. Photonics.

[50] Sönnichsen C., Franzl T., Wilk T., Plessen G., Feldmann J., Wilson O., Mulvaney P. (2002). Drastic reduction of plasmon damping in gold nanorods. Phys. Rev. Lett..

[51] Mackey M.A., Ali M.R., Austin L.A., Near R.D., El-Sayed M.A. (2014). The most effective gold nanorod size for plasmonic photothermal therapy: Theory and in vitro experiments. J. Phys. Chem. B.

